# Accuracy of sofa score evaluation and critical hyperglycemia after burns

**DOI:** 10.1186/2197-425X-3-S1-A583

**Published:** 2015-10-01

**Authors:** MK Belba, GP Belba

**Affiliations:** University Hospital Center Albania, Anesthesia and Intensive Care, Tirana, Albania; University Hospital Center Albania, Surgery, Tirana, Albania

## Introduction

The hyperglycemic condition from dysregulated glucose homeostasis has been defined stress hyperglycemia. Following burn trauma it persists in a hypermetabolic flow phase as a response of burn itself and sepsis. Serial Sequential Organ Failure Assesment (SOFA) score is recommended from American Burn Association for evaluation of organ dysfunction/failure.

## Objectives

The aim of this study is to test the relation of serial SOFA score evaluation and probability of critical hyperglycemia during burn injury.

## Methods

This is an observational prospective cohort study. Population is composed of adults hospitalized in ICU of the Service of Burns near University Hospital Center, Tirana, Albania for 5 years(2010-2015).Patients are grouped according glucose values on three categories: Patients with Euglycemia ( 80-120 mg/dL), Moderate Hyperglycemia (121-180 mg/dL) and Critical Hyperglycemia (> 180 mg/dL ).Test characteristics and performance as well as AUC are calculated for SOFA score on the 3^-rd^,7^-th^,14^-th^ and 21 ^-st^ day after burn injury.

## Results

**Figure 1:**
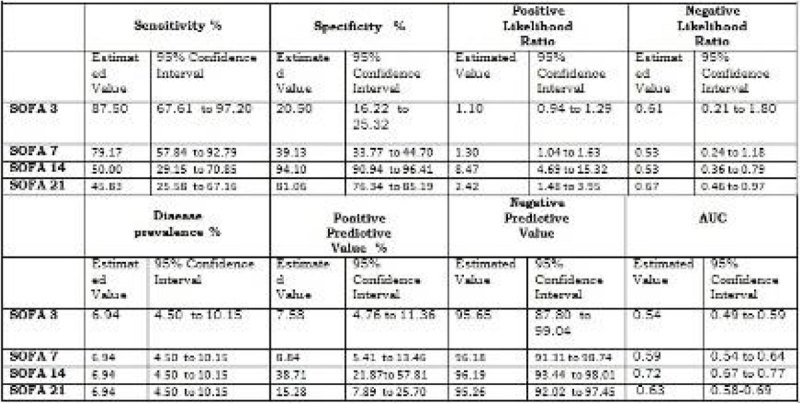
**Test performance-characteristics of SOFA score on 3, 7, 14, 21 day testing for critical hyperglycemia.**

**Figure 2:**
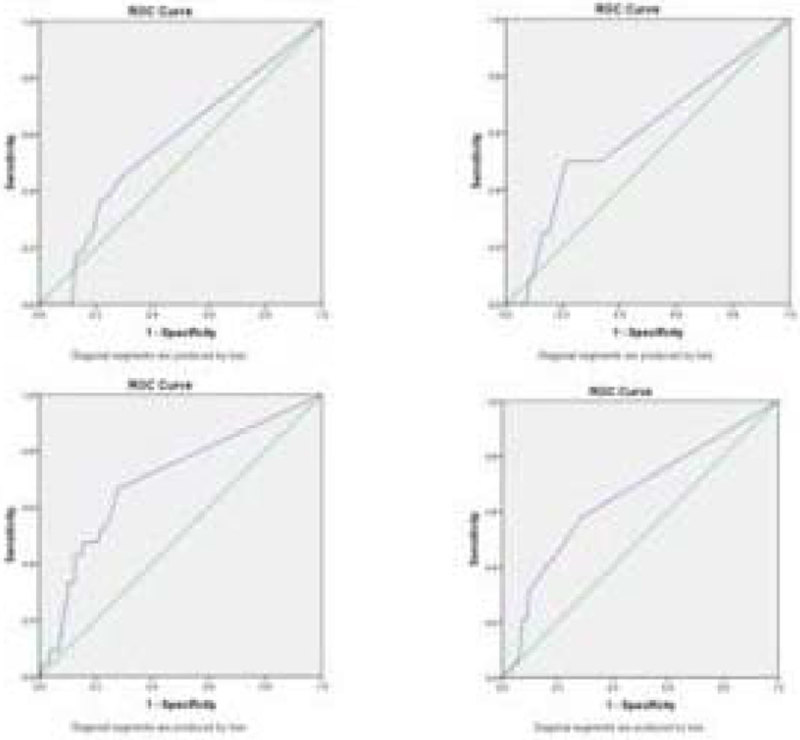
**Area Under the Curve for SOFA 3, 7, 14, 21 and Critical Hyperglycemia (Respectively: AUC 0.54;0.59;0.72;0.63)**

The prevalence of Critical Hyperglycemia in adult burn patients is 6.9%. Using the value 6 as the cutoff SOFA scoring for dysfunction/ failure, serial SOFA score evaluation and presence of critical Hyperglycemia have a good correlation. On the 14-th day after burn the values of Area Under the Curve, Positive Likelihood Ratios and Positive Predicted Values (PPV) are the better values as complementary information to clinical assessment (Figure 1, Figure 2).

## Conclusions

In patients with clinical sepsis after severe burns the probability for Critical hyperglycemia is higher after the first week (Positive Predicted Value 38.71% ) This tells us that 2 of 5 patients with sepsis after the first week of the illness will demonstrate critical hyperglycemia. Hyperglycemia is a sign of sepsis in severely burned adult patients.

